# Supplementary material for: Rosmarinic Acid, the Main Effective Constituent of Orthosiphon stamineus, Inhibits Intestinal Epithelial Apoptosis Via Regulation of the Nrf2 Pathway in Mice
Source: Molecules. 2019 Aug 21;24(17):3027. doi: 10.3390/molecules24173027 (PMC6749311; doi:10.3390/molecules24173027)
Supplement: Supplementary file 1 [file molecules-24-03027-s001.zip › S1-Materials and methods in detail.docx]

# S1 Materials and methods in detail

## Plant materials

*O. stamineus* was collected in Yulin (Guangxi province, China) in June, 2017. The plant was identified by Dr. H. B. Hu (Key Laboratory of Natural Drug Research and Development, Gannan Medical University) and a voucher specimen was retained in our laboratory for future reference (Voucher Number: OSL2017.06YL-002).

## Solvent Extraction and Partition Fractionation

*O. stamineus* was dried by under the direct sunlight(Abdullah et al., 2012). Dry powdered leaves (1000 g) were extracted with 50% aqueous ethanol using an ultrasonic extractor at 50^o^C for 30 min. The *O. stamineus* extracts were filtered and the concentrated liquid extract was freeze dried. The resulting powder was stored at −20°C until use. The condition was performed in triplicate. One hundred grams of finely milled freeze-dried extracts (OE) were redissolved with deionized water and petroleum ether, each using 1000 mL of the solvent (1:1), and then submitted to liquid-liquid partition. The petroleum ether phase was collected (PE). The aqueous phase were separated using molecular sieve chromatography method and the partition with a molecular weight that over 1000 (HE) and less than 1000 (but over 200) (LE) were collected respectively. All the fractions were freeze-dried and weighed up and evaluated for their total phenolics, total flavonoids and antioxidant activities. LE was then analyzed by HPLC to explore the major antioxidant compounds in OS.

## Contents of regular ingredients in O. stamineus and OE

The nutrient composition of the diets was determined using the standard Weende analysis methodology according to Chinese standard (GB/T 6432, GB/T 6433, GB/T 6435 and GB/T 6438). Protein in OE was measured by Coomassie brilliant blue method. Polysaccharide was analyzed by phonel-sulfate method. The total polyphenol content in the extracts was determined by the Folin–Ciocalteu method using gallic acid as the standard. Total flavonoids in the extract were determined with quercetin as the standard.

## Content of main comments in *Orthosiphon stamineus*

HPLC analyses were performed using an Acclaim Polarad vantage Ⅱ(4.6x150nm,3μm) at 25 ℃ with a mobile phase at a flow rate of 1.0 ml/min. The mobile phase consisted of 40% acetonitrile. The effluent was monitored at 254 - 340 nm (different standards using different wavelengths). Standard calibration curves were established by plotting the areas of peaks against different concentrations of the reference compounds (varying from 2.0 to 200 ng on column).

## Total phenolics and flavonoids analysis of O. stamineus extract

The total polyphenol content in the extracts was determined by the Folin–Ciocalteu method using gallic acid as the standard. Total flavonoids in the extract were determined with quercetin as the standard. Operate as described earlier(Cai et al., 2018).

## Reducing power and free radical scavenging activity of main comments in *Orthosiphon stamineus*

*Reducing power assay*

The ability of OE and main anioxidant to scavenge superoxide anion were determined according to the method of Ardestani et al(Ardestani and Yazdanparast, 2007) using assay kits according to the manufacturer’s instructions (Congyibio, Shanghai, China). The absorbance was measured at 700 nm. Increased absorbance of the reaction mixture correlates with greater reducing power.

*DPPH radical scavenging assay*

The DPPH radical scavenging activity of OE and main anioxidant were determined according to Ardestani et al(Ardestani and Yazdanparast, 2007) using assay kits according to the manufacturer’s instructions (Congyibio, Shanghai, China). The DPPH radical scavenging activity was calculated as follows:

DPPH radical scavenging activity (%) = (A *_test_* – A_PBS_) ×100/A_PBS_ .

*Superoxide anion scavenging activity assay*

The ability of OE and main anioxidant to scavenge superoxide anion were determined according to the method of Gow-Chin Yen et al. and Ardestani et al. (Ardestani and Yazdanparast, 2007; Yen and Chen, 1995) using assay kits according to the manufacturer’s instructions (Congyibio, Shanghai, China).. Superoxide radicals were generated in nicotineamide adenine dinucleotide, phenazine methosulphate (PMS–NADH) system by oxidation of NADH and assayed by reduction of nitroblue tetrazolium (NBT). The superoxide radical scavenging activity was calculated using the following formula:

Superoxide anion scavenging activity (%) = (A_PBS_ – A_test_)×100/A_PBS_

## Animals and treatment

Fifty male Balb/c mice weighing 18–20 g were purchased from Shanghai SLAC Laboratory Animal Co., Ltd. After being acclimated for 1 week, the mice were randomly divided into normal control (NC), high-fat control (FC), *O. stamineus* extracts (OE) and Rosmarinic acid (RA) groups, with ten mice in each group. The mice were housed in standard cages under controlled temperature conditions (22 ± 2 ℃) with a 12 h light/dark cycle. The NC group received only a normal diet (D12450B, Research Diet Inc.) containing 4.3% fat, and other groups received a high-fat diet (D12492, Research Diet Inc., New Brunswick, USA) containing 35% fat. The mice in the OE and RA groups were orally administered *OE or RA* at a dose of 100 mg/kg and 2 mg/kg body weight respectively, while the mice in the NC and FC groups were orally administered saline. The oral administration lasted for 8 weeks. At the end of the study, blood samples were collected by eyeball removal, and jejunal samples of mice were washed immediately with ice-cold PBS and stored at −80 ℃ prior to analysis. These experiments were carried out in accordance with local guidelines for the care of laboratory animals, and were approved by the institution’s ethics committee for research using laboratory animals.

## Measurement of Oxidative Stress in mice

Oxidative stress was assessed by measuring the GSH, Malondialdehyde (MDA) 8-hydroxy-2-deoxyguanosine (8-OHdG) content, 8-iso prostaglandin (8-iso-PG) content, glutathione peroxidase (GSH-Px), catalase (CAT), Glutathione S-transferase (GST) and superoxide dismutase (SOD) activity quantitatively. Jejunum homogenates were homogenized in 10 volumes (w/v) of ice-cold PBS with tissue homogenizer and centrifuged at 3000g for 15min at 4℃; then the supernatants were stored until the determination of antioxidant parameters. The activity of SOD was measured using the xanthine oxidase method, which monitored the inhibition of reduction of nitro blue tetrazolium in the sample. One unit of SOD activity was defined as the amount that reduced the absorbance at 550nm by 50% in 1 mg tissue homogenate protein and data were expressed as units/mg homogenates protein (U/mgprot). The activity of GSH-Px was detected with 5, 5’-dithiobisp-nitrobenzoic acid, and the change in absorbance at 412 nm was monitored. One unit of GSH-Px was determined as a decrease of 1mmol/l of GSH per 5 min for 1mg tissue homogenate protein at 37℃ after the subtraction of the non-enzymatic reaction. The activity of GST was detected at 37℃ with 1-chloro-2, 4-dinitrobenzene (CDNB) as the standard second substrate and reduced glutathione (GSH). One unit of GST was determined as a decrease of 1μmol/l of GSH per 1 min for 1mg tissue homogenate protein at 37℃ after the subtraction of the non-enzymatic reaction. The activity of CAT was measured using a spectrophotometric assay based on the formation of a stable complex with ammonium molybdate. MDA content in intestinal tissue in this study measured with 2-thiobarbituric acid (TBA) and expressed as nmol/mg protein. All the oxidative stress related items were measured using assay kits according to the manufacturer’s instructions (Nanjing Jiancheng Bioengineering Institute). 8-OHdG and 8-iso-PG were detected in ELISA way and the assay kit purchased from Cusabio (Wuhan, China) and Shanghai Enzyme-linked Biotechnology (Shanghai, China) respectively. The protein content of homogenates was measured using the coomassie brilliant G-250 method.

## Quantitative real-time PCR analysis

Total mRNA was extracted from the intestinal epithelial tissue with TransZol Up reagent (Transgen Biotech, Beijing, China) and then immediately reverse transcribed to cDNA according to the manufacturer’s instructions (Takara, Japan). The qualities of total RNA were assessed using both Nanodrop Lite (1.8 ≤ A260/280 ≤ 2.0 believed high qualities, Thermo, USA) and 1.5% agarose gel. A real-time PCR assay was used to quantify gene expression. The primer sequences are shown in Table 1. The reactions for each sample were performed in triplicate, and each 20-μl reaction mixture included 1 μl of cDNA, 0.4 μl of forward primer, 0.4 μl of reverse primer, 8.2 μl of ddH_2_O, and 10 μl of SYBR Premix Ex Taq. The reactions were incubated in an ABI 7500 system at 95℃ for 30s, followed by 40 cycles of denaturation at 95℃ for 5s, annealing and extension at 60℃ for 30s.

Gene expression was quantified using real-time qPCR analyzer software by ABI Company. The relative expression of mRNA species was determined using the comparative Ct method (Chueh and Lin).

Table 1. Sequences of primers used for real-time PCR

| Gene name | Primer(5’→3’) | References |
| --- | --- | --- |
| Nrf2 | F: CGAGATATACGCAGGAGAGGTAAGA  R: GCTCGACAATGTTCTCCAGCTT | (Li et al., 2016) |
| HO-1 | F:CCTCACTGGCAGGAAATCATC  R: CCTCGTGGAGACGCTTTACATA | (Wu et al., 2014) |
| NQO1 | F: TTCTGTGGCTTCCAGGTCTT  R:AGGCTGCTTGGAGCAAAATA | (Li et al., 2016) |
| GST | F:AAGACCACAGCACCAGCACCAT  R: CTCTCCTCCTCTGTCTCTCCAT | (Cho et al., 2002) |
| SOD1 | F: TGGAGACCTGGGCAATGTGACT  R: TCCACCTTTGCCCAAGTCATCT | (Wang et al., 2015) |
| GAPDH | F: CTTCACCACCATGGAGAAGGC  R: GGCATGGACTGTGGTCATGAG | (Sethi et al., 2012) |

## Electrophoretic mobility-shift assay (EMSA)

Nuclear Nrf-2 DNA binding activity was measured by EMSA using the EMSA kit (Thermo Fisher). The assay was performed in accordance with manufacturer’s instructions. Oligonucleotide corresponding to the binding sites of ARE nucleotide. The sequences of oligonucleotides can be found as follows:

The sequences of cold competitor oligonucleotide

| Name | Sequence(5’→3’) |
| --- | --- |
| Labeled Probe (F) | ACTGAGGGTGACTCAGCAAAATC |
| Labeled Probe (R) | GATTTTGCTGAGTCACCCTCAGT |
| Cold competitive probe (F) | ACTGAGGGTGACTCAGCAAAATC |
| Cold competitive probe (R) | GATTTTGCTGAGTCACCCTCAGT |
| Mutant competitive probe (F) | ACTGAGGGTGACTCAATAAAATC |
| Mutant competitive probe (R) | GATTTTATTGAGTCACCCTCAGT |

The specificity of the band was confirmed by competition with cold oligonucleotides and mutated oligonucleotides. Gels were imaged using the scanner (V300, Epson, Japan) followed analyzed by alphaEaseFC system (Alpha Innotech, Portugal).

The binding reaction consisted as follows:

Unit: μl

| Regent | Negative control | Sample | Competition control |
| --- | --- | --- | --- |
| 10×binding buffer | 2 | 2 | 2 |
| 1ug/ul poly（dl.dc） | 1 | 1 | 1 |
| 50％glycerol | 1 | 1 | 1 |
| 1%NP-40 | 1 | 1 | 1 |
| 100m M Mgcl | 1 | 1 | 1 |
| 200m M EDTA | 1 | 1 | 1 |
| Protein | - | + | + |
| labeled probe (200 fmol) | + | + | + |
| unlabeled probe (40 pmol) | - | - | + |
| H_2_O | + | + | + |
| Total | 20μl | 20μl | 20μl |

## Measurement of villus height and crypts depth

Jejunum tissues of mice were collected and processed as per standard protocol. Briefly, the tissues were fixed in 4% neutral buffered formalin and then embedded in paraffin wax, sectioned at 5–10 µm in rotary microtome and stained with hematoxylin and eosin (HE). The villus and crypts were measured by using the segmented line tool. The heights of villus were calculated from the base of the villus, above the level of adjoining crypts, to the villus tip. The depths of crypts were calculated from the boundaries between crypts and myenteron to the base of the villus. At least 10 typical villus and their crypts were measured for each animal and the mean value calculated.

## DNA fragmentation analysis

A TUNEL kit (Boster, Wuhan, China) was used for DNA fragmentation analysis. All operations conducted accorded to the manufacturer’s protocol. For each slide, 5 field were randomly chosen under the microscope on each section. The apoptotic index was determined by dividing the number of apoptotic cells by the total number of cells in the crypt column and multiplying by 100.

## Caspase -3, -8 and -9 activities Analysis

Activities of Caspase -3, -8 and -9 were measured using substrate peptides Ac-DEVD-pNA, Ac-IETD-pNA and Ac-LEHD-pNA by Caspase activity kits (Beyotime, Shanghai, China). All operations conducted accorded to the manufacturer’s protocol. One unit is the amount of enzyme that will cleave 1.0nmol of the colorimetric substrate Ac-DEVD-pNA, Ac-IETD-pNA or Ac-LEHD-pNA respectivly per hour at 37ºC under saturated substrate concentrations.

## Western blot analysis

Jejunum tissue were homogenized in a lysis buffer with 20mM Tris (pH7.5)，150mM NaCl and Triton X-100. The insoluble material was removed by centrifugation at 10, 000 g, 4 °C for 5 min and the supernatants were collected.

For Nrf2 test, the nuclear extracts of mice jejunum were prepared using a nuclear protein Extraction kit (Beyotime, Shanghai, China).

Protein concentration was determined with BCA assay kit (Beyotime, Shanghai, China). Samples were electrophoresed using 10% SDS-PAGE, transferred onto polyvinylidene fluoride membranes and then incubated with specific primary antibodies. After the membranes had been washed, the membranes were incubated with the horseradish peroxidase–linked secondary antibody. The density of the band was quantified with enhanced chemiluminescence (ECL) reagent and detected by a gel analysis system. All the antibodies in this study were purchased from Boster Biological Technology (Wuhan, China) or Cell Signaling Technology Inc (Danvers, USA) : FAS-L Antibody, Catalog #PB0042; Cytochrome C, Catalog #BA0781; CASP3(P17) Antibody Antibody, Catalog #BA3257<CST#9662>; CASP8<CST#9746>; CASP9<CST#9504>; Nrf2< CST#12721 >.

## Statistical analysis

All reaction mixtures were prepared in triplicate and at least three independent assays were performed for each sample. All data are expressed as mean ± SEM. Data were subjected to one-way ANOVA followed by Duncan’s multiple range tests using SPSS version 17.0 software. A P value < 0.05 was considered to be statistically significant.

## Refrence

Abdullah, S., Shaari, A.R., Azimi, A., 2012. Effect of Drying Methods on Metabolites Composition of Misai Kucing (Orthosiphon stamineus) Leaves, in: Dan, Y. (Ed.) 3rd International Conference on Biotechnology and Food Science. pp. 178-182.

Ardestani, A., Yazdanparast, R., 2007. Antioxidant and free radical scavenging potential of Achillea santolina extracts. Food Chemistry 104(1), 21-29.

Cai, X., Xiao, C., Xue, H., Xiong, H., Hang, Y., Xu, J., Lu, Y., 2018. A comparative study of the antioxidant and intestinal protective effects of extracts from different parts of Java tea (Orthosiphon stamineus). Food Science & Nutrition 6(3), 579-584.

Cho, H.-Y., Jedlicka, A.E., Reddy, S.P.M., Kensler, T.W., Yamamoto, M., Zhang, L.-Y., Kleeberger, S.R., 2002. Role of NRF2 in Protection Against Hyperoxic Lung Injury in Mice. American Journal of Respiratory Cell and Molecular Biology 26(2), 175-182.

Chueh, W.H., Lin, J.Y., 2011. Berberine, an isoquinoline alkaloid, inhibits streptozotocin-induced apoptosis in mouse pancreatic islets through down-regulating Bax/Bcl-2 gene expression ratio. Food Chemistry 132, 252-260.

Li, J., Sapper, T.N., Mah, E., Rudraiah, S., Schill, K.E., Chitchumroonchokchai, C., Moller, M.V., McDonald, J.D., Rohrer, P.R., Manautou, J.E., Bruno, R.S., 2016. Green tea extract provides extensive Nrf2-independent protection against lipid accumulation and NFκB pro- inflammatory responses during nonalcoholic steatohepatitis in mice fed a high-fat diet. Molecular Nutrition & Food Research 60(4), 858-870.

Sethi, J., Sanchez-Alavez, M., Tabarean, I.V., 2012. Loss of histaminergic modulation of thermoregulation and energy homeostasis in obese mice. Neuroscience 217(0), 84-95.

Wang, D., Wang, Y., Wan, X., Yang, C.S., Zhang, J., 2015. Green tea polyphenol (−)-epigallocatechin-3-gallate triggered hepatotoxicity in mice: Responses of major antioxidant enzymes and the Nrf2 rescue pathway. Toxicology and Applied Pharmacology 283(1), 65-74.

Wu, Q., Zhang, D., Tao, N., Zhu, Q.-N., Jin, T., Shi, J.-S., Liu, J., 2014. Induction of Nrf2 and Metallothionein as a Common Mechanism of Hepatoprotective Medicinal Herbs. The American Journal of Chinese Medicine 42(01), 207-221.

Yen, G.-C., Chen, H.-Y., 1995. Antioxidant Activity of Various Tea Extracts in Relation to Their Antimutagenicity. Journal of Agricultural and Food Chemistry 43(1), 27-32.
