# Supplementary material for: Rosmarinic Acid, the Main Effective Constituent of Orthosiphon stamineus, Inhibits Intestinal Epithelial Apoptosis Via Regulation of the Nrf2 Pathway in Mice
Source: Molecules. 2019 Aug 21;24(17):3027. doi: 10.3390/molecules24173027 (PMC6749311; doi:10.3390/molecules24173027)
Supplement: Supplementary file 1 [file molecules-24-03027-s001.zip › S2-HPLC analysis of main content in EE.docx]

S2 HPLC analysis of main content in EE

This document summarized HPLC chromatogram for EE and the standard substances. All the chromatogram acquired with uniform conditions (described in S1) and the content of main phenolic acids and flavonoids compounds were quantified by standard curve method.


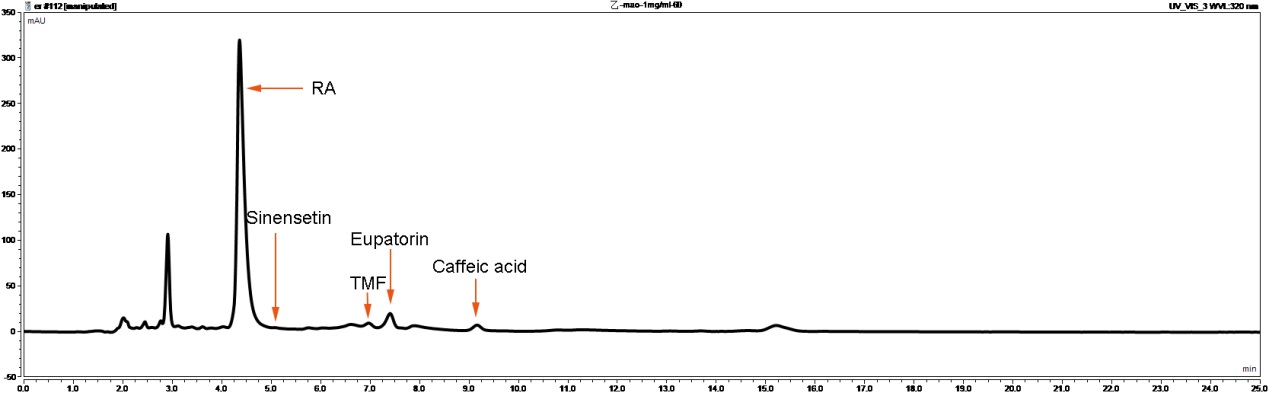


S2-1 HPLC chromatogram for EE.


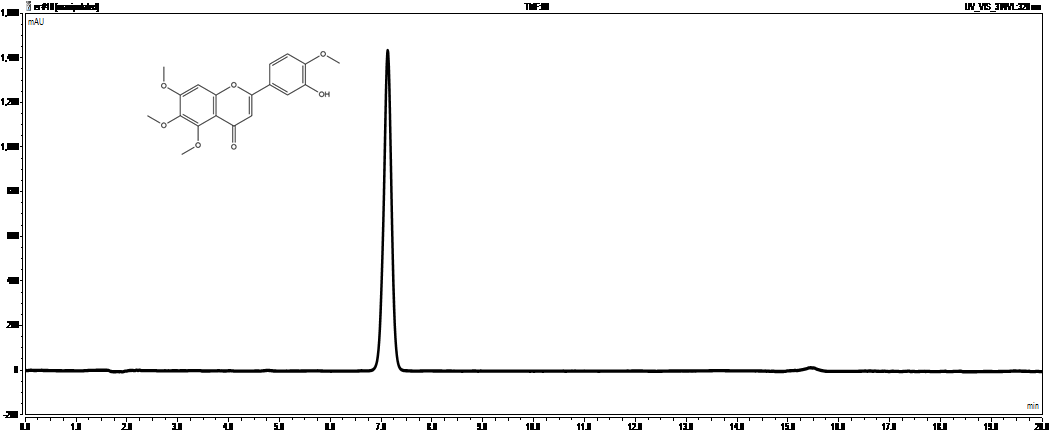


S2-2 HPLC chromatogram for TMF.


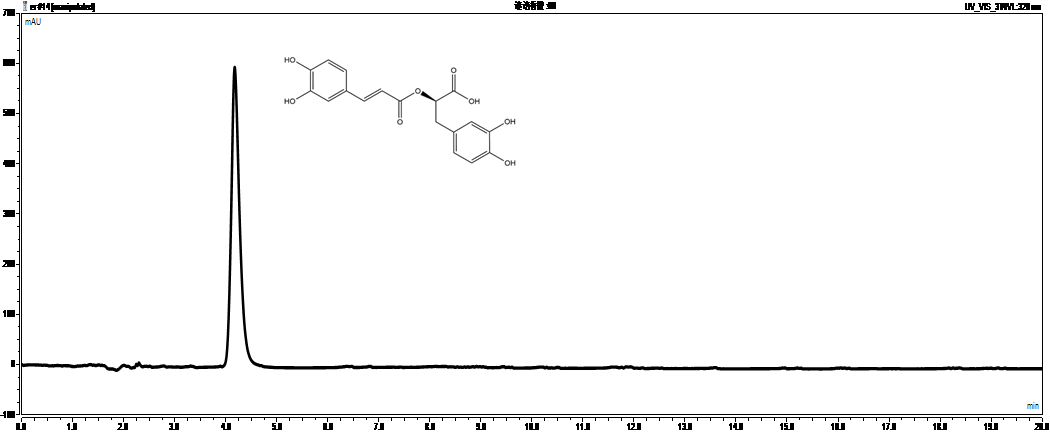


S2-3 HPLC chromatogram for RA.


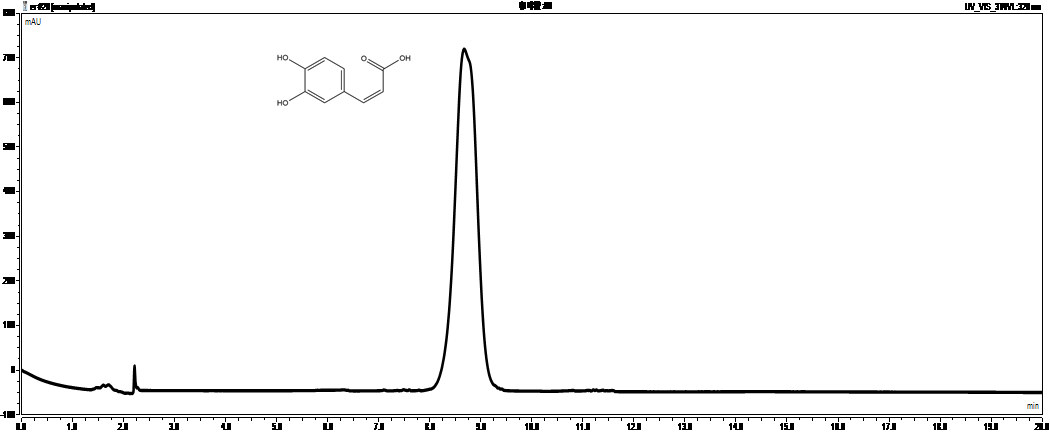


S2-4 HPLC chromatogram for caffeic acid.


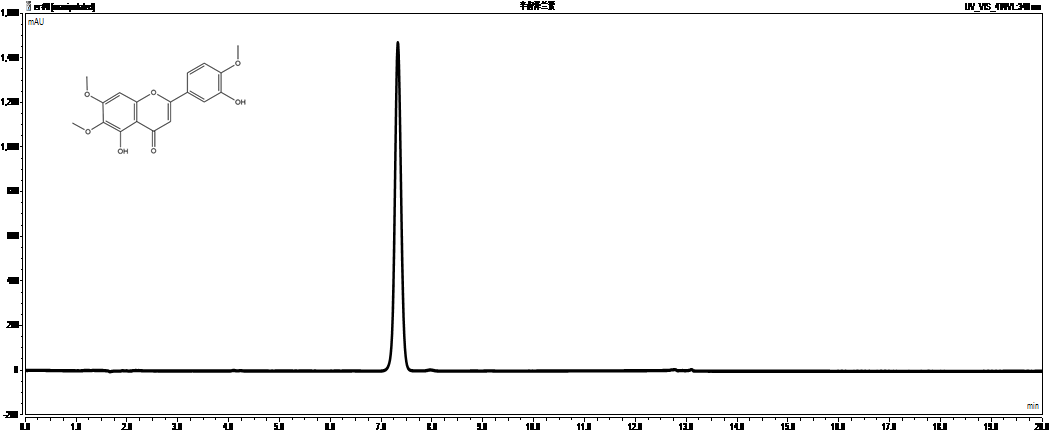


S2-5 HPLC chromatogram for eupatorin.

­
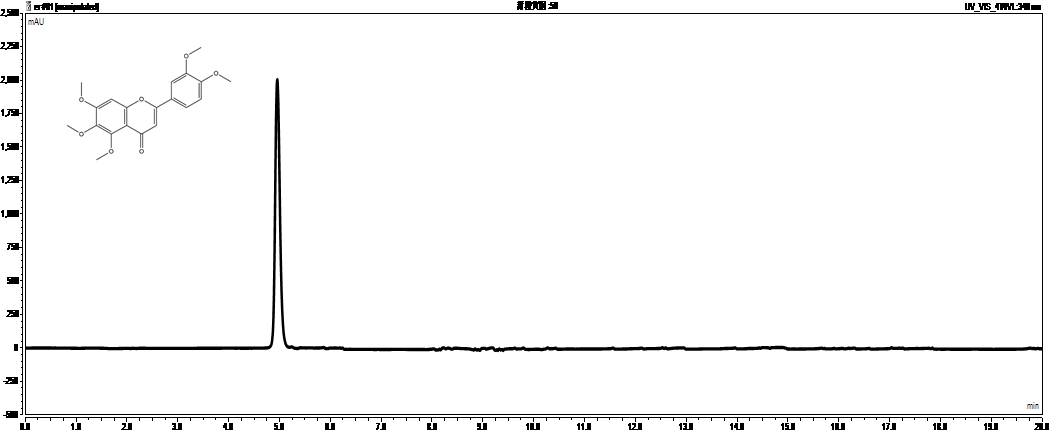


S2-6 HPLC chromatogram for sinensetin.
