# Supplementary material for: Rosmarinic Acid, the Main Effective Constituent of Orthosiphon stamineus, Inhibits Intestinal Epithelial Apoptosis Via Regulation of the Nrf2 Pathway in Mice
Source: Molecules. 2019 Aug 21;24(17):3027. doi: 10.3390/molecules24173027 (PMC6749311; doi:10.3390/molecules24173027)
Supplement: Supplementary file 1 [file molecules-24-03027-s001.zip › S3 - Body weight and blood glucose of mice.docx]

# S3 Body weight and blood glucose of mice


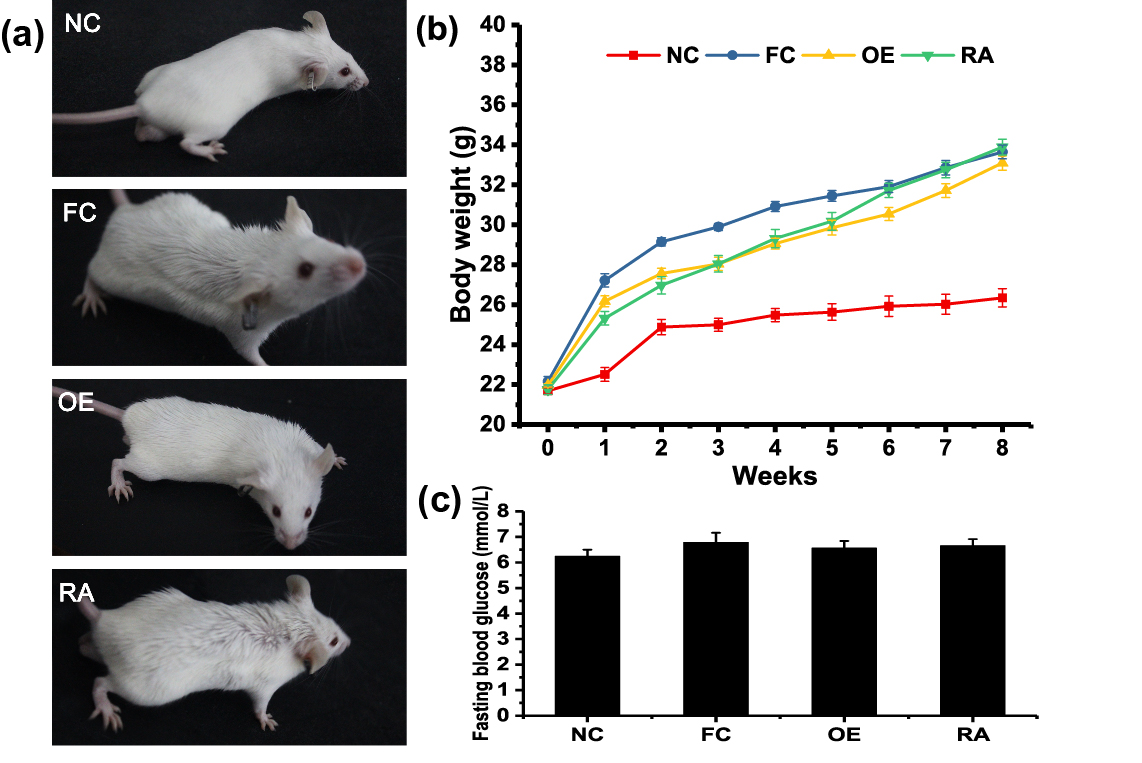


Fig. S3 Body weight and blood glucose of mice. (a) The mice were photographed at the end of 8 weeks. (b) The mice were weighted weekly and no significantly difference between FC, OE and RA groups were observed at the end of week 8 (*p* > 0.05); but these three groups significantly heavier than NC group (*p* < 0.05). (c) All the mice were fasting for 12h before sacrificed. Blood glucose was measured by glucose oxidase-peroxidase method. No significantly difference observed (*p* > 0.05), but the blood glucose in FC group has a trend higher than that in NC group (*p* < 0.1).
