# Supplementary figures and images for: Rosmarinic Acid, the Main Effective Constituent of Orthosiphon stamineus, Inhibits Intestinal Epithelial Apoptosis Via Regulation of the Nrf2 Pathway in Mice
Source: Molecules. 2019 Aug 21;24(17):3027. doi: 10.3390/molecules24173027 (PMC6749311; doi:10.3390/molecules24173027)

S4- High magnification figures about HE stain


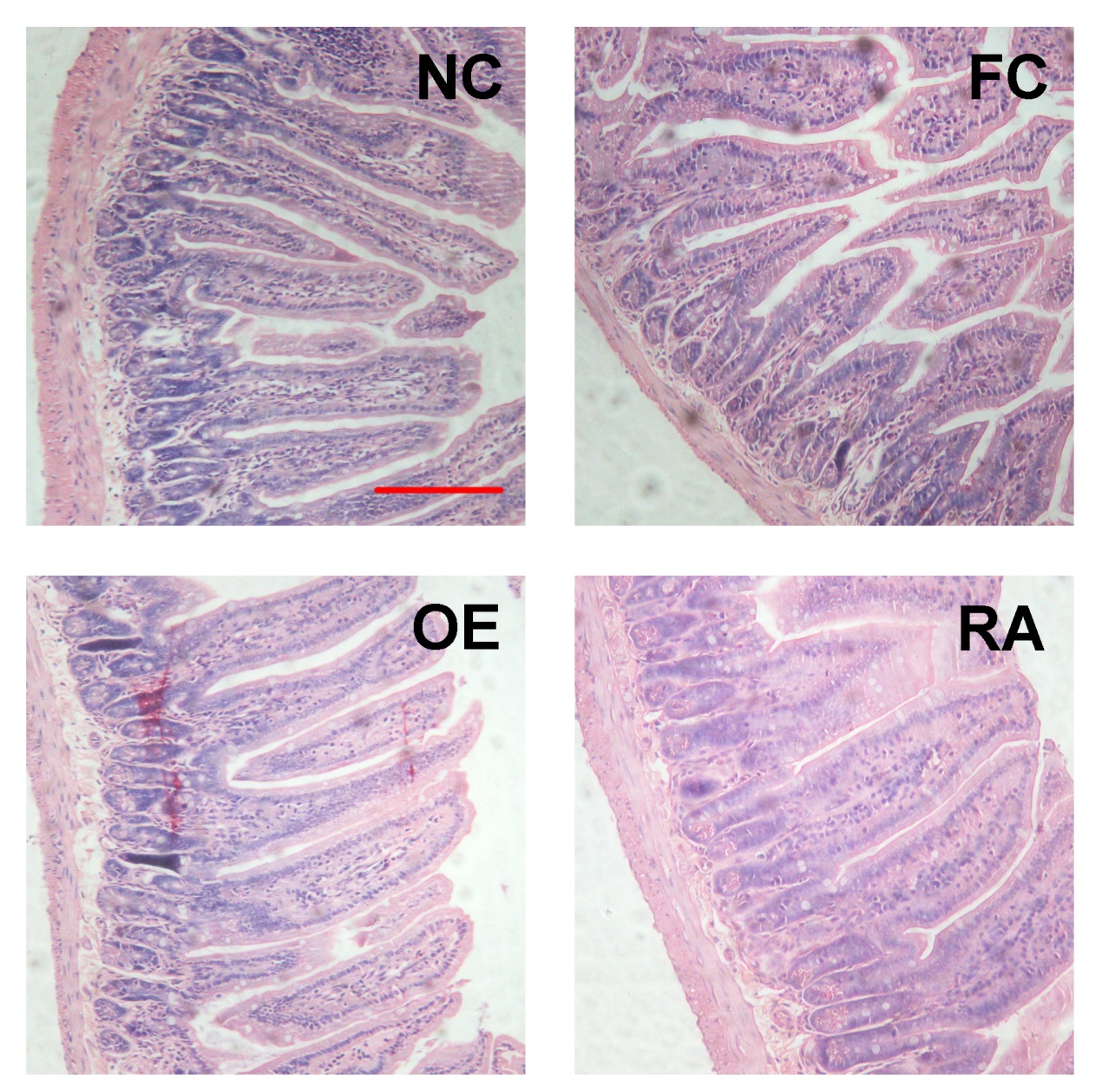


HE stain by 200X magnification, bar = 100μM.

Supplement: Supplementary file 1 [file molecules-24-03027-s001.zip › S4- High magnification figures about HE stain.docx]
